# Study of *mcr-1* Gene-Mediated Colistin Resistance in Enterobacteriaceae Isolated from Humans and Animals in Different Countries

**DOI:** 10.3390/genes8120394

**Published:** 2017-12-19

**Authors:** Linda Hadjadj, Toilhata Riziki, Yan Zhu, Jian Li, Seydina M. Diene, Jean-Marc Rolain

**Affiliations:** 1 Unité de Recherche sur les Maladies Infectieuses et Tropicales Émergentes (URMITE), UMR CNRS, IHU Méditerranée Infection, Faculté de Médecine et de Pharmacie, Aix-Marseille-University, Marseille 13005, France; linda.hadjadj@univ-amu.fr (L.H.); t.riziki@yahoo.com (T.R.); seydina.m.ddiene@gmail.com (S.M.D.); 2 Monash Biomedicine Discovery Institute, Department of Microbiology, Monash University, Parkville, Victoria 3800, Australia; yan.zhu@monash.edu (Y.Z.); jian.li@monash.edu (J.L.)

**Keywords:** *mcr-1*, colistin, genome, integration, IS*Apl1*, plasmid

## Abstract

In this study, we aim to characterize the genetic environment of the plasmid-mediated colistin resistance gene *mcr-1* in 25 *Escherichia coli* and seven *Klebsiella pneumoniae* strains from different countries and continents. Multilocus sequence typing, conjugation experiments, plasmid typing, and the presence and location of the insertion sequence IS*Apl1* were investigated. Whole genome sequencing of four *E. coli* was performed to analyse the genetic environment of the *mcr-1* gene. Colistin minimum inhibitory concentration of *mcr-1* strains varied from 3 to 32 µg/mL. Six *E. coli* sequence types were detected: ST 4015, ST 3997, ST 10, ST 93, ST 48, and ST 648. IncHI2, IncI2, and IncP plasmid types were predominant and were unrelated to a specific country of origin. IS*Apl1* was found in 69% of analysed plasmids that were mainly around the *mcr-1* gene. Analysis of four closed *mcr-1* plasmids revealed the integration of *mcr-1* into hotspots. We found that the spread of *mcr-1* gene was due to the diffusion of a composite transposon and not to the diffusion of a specific plasmid or a specific bacterial clone. The ease with which the *mcr-1* gene integrates into various regions facilitates its dissemination among bacteria and explains its large diffusion all over the world, both in animals and in humans.

## 1. Introduction

Antibiotic resistance is a major issue around the world. This phenomenon has led clinicians to adapt treatment strategies and to use powerful, broad spectrum antibiotics, such as carbapenems against multi drug resistant Gram-negative bacteria. However, the recent emergence of carbapenemase-producing bacteria around the world [1] has obliged clinicians to turn, as a last resort, to colistin [2,3]. The re-use of colistin has led to the appearance of colistin resistance, which is mediated by complex chromosomal resistance mechanisms in human and animal isolates [4,5]. Recently, a transferable colistin resistance mechanism, due to the presence of *mcr-1* genes and variants that code for a phosphoethanolamine transferase, which have been detected in all continents [5] on a plasmid, has been described [6,7,8,9,10,11]. Initially, the *mcr-1* gene was carried on IncI2-type plasmid, but has also been found in other plasmid types, such as IncHI2, IncX4, and IncP [5,12,13]. Generally, the *mcr-1* gene has been described as being associated with an open reading frame (ORF), encoding a protein that is similar to a PAP2 superfamily protein, following the *mcr-1* gene with an insertion sequence IS*Apl1* downstream to it [6]. IS*Apl1* is an insertion sequence that belongs to the IS30 family of transposons and was initially described in *Actinobacillus pleuropneumoniae* [14]. The *mcr-1* gene may be surrounded by two copies of IS*Apl1*, leading to the formation of a composite transposon. This transposon Tn*6330* (IS*Apl1*-*mcr-1*-ORF-IS*Apl1*) has been described as a composite transposon that is able to mobilize the *mcr-1* gene [15,16]. However, to date, the integration of this transposon into plasmids, its stabilization, and its evolution have not been comprehensively described. Thus, the aim of our study was to conduct an epidemiological and molecular characterization of *mcr-1* strains from different origins, and to study the genetic environment of the *mcr-1* gene and to characterize four different plasmids mediating colistin resistance for a better understanding of the integration of the *mcr-1* gene into plasmids.

## 2. Materials and Methods

### 2.1. Strains

Thirty-two *mcr-1* positive strains, including 25 *Escherichia coli* and seven *Klebsiella pneumoniae*, from Laos, Thailand, France, and Algeria, as well as from Hajj pilgrims (returning from their pilgrimage from Saudi Arabia to France), were analysed [17,18,19,20,21,22]. Five isolates were from animals and 27 from humans (Table 1). The minimum inhibitory concentration (MIC) of colistin was tested by microdilution in accordance with European Committee on Antimicrobial Susceptibility Testing (EUCAST) recommendations [23]. The presence of the *mcr-1* gene was confirmed by real-time polymerase chain reaction (RT-PCR) [24] and multilocus sequence typing performed using the Warwick method [25] on *E. coli* strains and the Pasteur method [26] on *K. pneumoniae* strains. Resistance stability was analysed by subculture over the course of 30 passages of each strain and by checking for the presence of the *mcr-1* gene every five passages. In the event of gene loss, the MIC of colistin was tested and the procedure was repeated to confirm the results.

### 2.2. Conjugation and Plasmid Analysis

Conjugation was tested with azide-resistant *E. coli* J53. Transconjugants were selected on MacConkey agar (Beckton Dickinson, Le Pont de Claix, France) added by 120 µg/mL sodium azide and 4 µg/mL colistin, as described [27]. In the event of unsuccessful conjugation, transformation using the electroporation method was performed [28]. Transformants bacteria were selected on Luria Bertani agar (Beckton Dickinson, Le Pont de Claix, France) supplemented with 4 µg/mL colistin (Sigma-Aldrich, Saint Louis, USA). The presence of the *mcr-1* gene was tested by RT-PCR on transconjugant and transformant strains. Plasmid typing was carried out on positives. Nineteen different plasmid types, including IncI2 [29,30], and the presence of the IS*Apl1* insertion sequence both downstream and upstream of the *mcr-1* gene were tested by conventional PCR [31].

### 2.3. Whole Genome Analysis

Four *E. coli* strains (1RC4, LH1, LH30, LH57), one isolated from Hajj pilgrims, and three from healthy individuals living in Laos (Table 1), were sequenced using the Next Generation Sequencing (NGS) Miseq (Illumina Inc., San Diego, CA, USA). We selected the strain *E. coli* LH1 because conjugation and transformation experiments failed and we suspected a chromosomal location of *mcr-1* gene. The strain *E. coli* LH30 was chosen to understand the rapid loss of *mcr-1* gene in the resistance stability experiment. We chose the strain *E. coli* LH57 because a chromosomal mechanism of colistin resistance was described in this strain. The strain *E. coli* 1RC4 was acquired during Hajj pilgrims, and we found the origin of this plasmid. Genomes were assembled using the A5 pipeline, annotated by RAST [32], resistance gene by ARG-ANNOT [33], plasmid presence by Plasmid finder software, and plasmid multilocus sequence typing by pMLST software [34]. The percentage of similarity between plasmids was calculated using a pairwise comparison of their Average Nucleotide Identity based on Blast (ANIb) [35] and using the Jspecies software [36].

A database containing all *mcr-1* complete plasmids that were available in the National Center for Biotechnology Information (NCBI) database as of 14 February 2017 was created (Table 2). Plasmid type was then determined using the Plasmid finder software, and in-silico analysis of *tra* genes was performed for the non-conjugative *E. coli* LH1 strain. The presence of insertion sequence IS*Apl1* in all plasmids, including those that were present in our created database, was checked. Our four complete *mcr-1* plasmids were compared and visualized using CGView software [37]. These plasmids were named pLH30-mcr1, pLH57-mcr1, pLH1-mcr1, and p1RC4-mcr1, and were submitted to Genbank under accession numbers NKYL00000000, NKYM00000000, NKYJ00000000, and NKYK00000000, respectively. An analysis of the genetic environment of the *mcr-1* gene was performed using blast X on all of the genes around the *mcr-1* gene in the NCBI. The identity of insertion sequences was confirmed using the ISfinder software [38].

## 3. Results

### 3.1. Strain Characteristics

**Figure 1:**
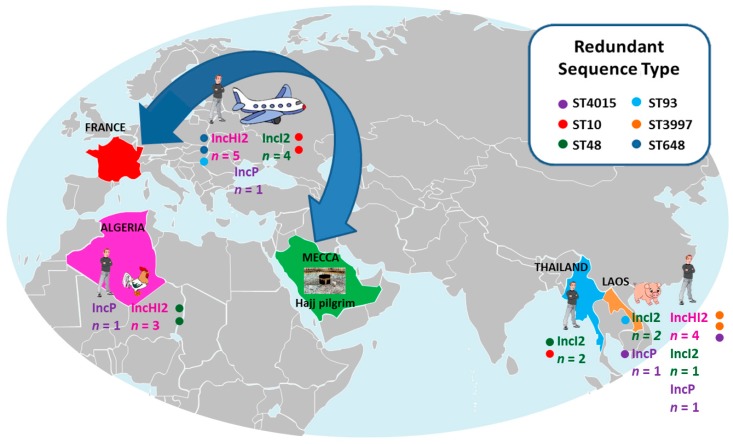
Origins and plasmid type of *mcr-1* strains collected in our study.

The colistin MIC of strains harboring the *mcr-1* gene varied from 3 to 32 µg/mL (Table 1). The presence of known chromosomal mechanisms of colistin resistance was found in two *E. coli* strains due to the PhoQ mutation (E375K) [21]. Regarding three *K. pneumoniae* strains, resistance was due to mgrB stop in the first strain, a mgrB substitution in the second, and a PmrB mutation (T157P) in the last [22] (Table 1). Different clones were identified with seven different sequence types (STs) in *K. pneumoniae* strains and 17 different STs in *E. coli* strains, including six recurrent STs, namely ST 4015, ST 3997, ST 10, ST 93, ST 48, and ST 648 (Table 1, Figure 1). Following the subculture of strains, the loss of the *mcr-1* gene was observed in four *K. pneumoniae* strains after 25 passages. For *E. coli* strains, only LH30 lost its *mcr-1* gene after 20 passages. Colistin susceptibility was restored in *E. coli* LH30 upon the loss of *mcr-1* gene. However, this was not the case for *K. pneumoniae* strains, which continued to be colistin resistant after the loss of *mcr-1* (Table 1).

### 3.2. Location of the mcr-1 Gene

The presence of plasmids was demonstrated in all 25 *E. coli* strains, of which 21 carried the *mcr-1* gene on a conjugative plasmid. For *K. pneumoniae* strains, only two carried the *mcr-1* gene on a conjugative plasmid (Table 1). The predominance of IncHI2-type (44.5%), IncI2-type (40.7%), and IncP-type (14.8%) plasmids was observed on the whole strains. The majority of plasmids (77.8%) were isolated from humans, including 81.8% of IncI2-type, 75% of IncHI2-type, and 75% of IncP-type plasmids (Table 1). The presence of insertion sequence IS*Apl1* around the *mcr-1* gene was found in 56.25% of strains in the downstream position, and 12.5% in the downstream and upstream positions. Furthermore, 28.12% of strains did not have an IS*Apl1* insertion sequence that was close to the *mcr-1* gene, and 3.12% had partial IS*Apl1* sequences in positions that were downstream and upstream of the *mcr-1* gene.

### 3.3. Genome Analysis

#### *Mcr-1* Database

**Figure 2:**
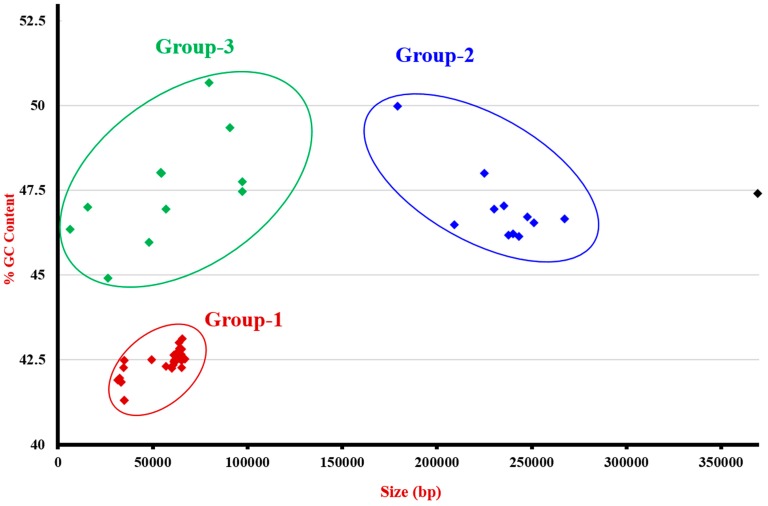
Diversity of plasmids carrying the *mcr-1* gene. All of these plasmids were retrieved from the NCBI database and were plotted according to their GC% and size. Group-1 includes plasmids from *E. coli*, *C. sakazaki*, *K. pneumoniae*, *K. ascorbata*, *S. enterica*, and *S. sonnei.* The average size and %GC were estimated as 51.88-Kb and 41.32 GC%, respectively. The identified incompatibility plasmid types were IncI2, IncX4. Group-2 includes plasmids from *E. coli* only. The average size and %GC content were 244.81-Kb and 47.02 %GC, respectively. The incompatibility plasmid types of this group were: IncHI1B, IncHI2, IncFIB, IncN. Group-3 includes plasmids from *E. coli*, *K. pneumoniae*, and *S. enterica.* Their sizes varied from 6. 39 to 97.56-Kb and the GC% content from 44.89 to 50.57%. The incompatibility plasmid types of this group were IncFII, IncX4, IncFIB, IncY. A single plasmid from *E. coli* (in black) with size of 369.30-Kb, 47.4% of GC content, and belonging to the IncN plasmid type was identified.

Fifty-nine *mcr-1* complete plasmids were retrieved from the NCBI database. Taking into account the % GC content and the plasmid sizes, our database can be divided into three main groups. The plasmid size was 51.88 Kb in group 1, 244.81 Kb in group 2, and 6.39–97.56 Kb in group 3 (Figure 2). Our created *mcr-1* plasmid database enabled us to see that the closest *mcr-1* plasmid for pLH30-mcr1 was KU743384, with 99.68% similarity, while pLH57-mcr1 was KX276657, with 99.11% similarity, pLH1-mcr1 was KX254341, with 99.91% similarity, and p1RC4-mcr1 was KU743384, with 99.98% similarity. All of these plasmids were isolated from *E. coli* strains. KU743384 was an IncHI2 plasmid that was isolated from *E. coli* ST68 in Saudi Arabia. KX276657 was an Inc F18:A-:B1 (IncN with Plasmid finder software) plasmid isolated from *E. coli* ST457 in the United States, while KX254341 was an IncHI2 plasmid also isolated from *E. coli* in China (Table 2). From our *mcr-1* database, 49% of plasmids had IS*Apl1* around the *mcr-1* gene, of which 75.9% were in a downstream position and 24.1% were transposons Tn*6330* (Table 2). In eight plasmids, we also found a copy of IS*Apl1* sequences around the *mcr-1* gene, as well as in other plasmid locations. For 51% of plasmids, no IS*Apl1* sequence was detected in the whole plasmid. The majority of plasmids were isolated from *E. coli* strains. Plasmid sizes ranged from 33 Kb to 369 Kb, and GC content varied from 41.84% to 50.67%. Eight types of plasmids were represented in the database, including 40.7% IncI2 and 25.4% IncX4 (Figure 2, Table 2).

### 3.4. Sequenced Plasmids 

Concerning the four plasmids that we sequenced, plasmid sizes ranged from 219 Kb to 248 Kb, and GC% ranged from 46 to 48. The plasmids were all IncHI2 type. pLH30-mcr1 and pLH1-mcr1 belonged to ST 3, while p1RC4-mcr1 was ST 1 and pLH57-mcr1 had an unknown ST (Table 3). A single copy of the *mcr-1* gene was found in all of our plasmids. Antibiotic resistance genes other than *mcr-1* are shown in Table 3. On pLH1-mcr1, the IS*Apl1* sequence was found to be inserted into the *traE* gene, leading to a stop codon that inactivated it.

### 3.5. Genetic Integration of mcr-1 Gene

**Figure 3:**
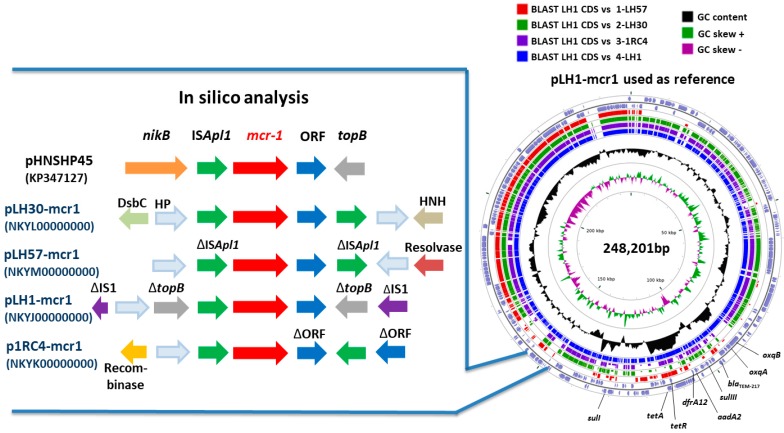
Genetic environment of the *mcr-1* gene in plasmids: pHNSHP45 (KP347127), pLH30-mcr1 (NKYL00000000), pLH57-mcr1 (NKYM00000000), pLH1-mcr1 (NKYJ00000000), and p1RC4-mcr1 (NKYK00000000). Comparison of the four complete *mcr-1* plasmids was performed using blastP with CGview software. *nickB* refers to Nickel B, *topB*: topoisomerase B, DsbC: Thiol:disulfide interchange protein DsbC, HP: Hypothetical protein, HNH: HNH endonuclease, IS: Insertion sequence.

In silico analysis confirmed the presence of IS*Apl1* downstream and upstream of the *mcr-1* gene in the pLH30-mcr1. The transposon was surrounded by two genes coding for hypothetical proteins, with one gene coding for a thiol:disulfide interchange protein DsbC to the left and one coding for a HNH endonuclease to the right of the *mcr-1* gene. In p1RC4-mcr1, we observed an IS*Apl1* sequence downstream and upstream in opposite directions, as well as two truncated ORFs and a recombinase close to the *mcr-1* gene. In pLH1-mcr1, a classic *mcr-1* cassette (IS*Apl1*-*mcr-1*-ORF) and truncated IS1 insertion sequences were found around the *mcr-1* transposon. For pLH57-mcr1, we observed a truncated IS*Apl1* sequence downstream and upstream, and a resolvase near the *mcr-1* transposon (Figure 3).

## 4. Discussion

The presence of the *mcr-1* gene led to a relatively low colistin resistance between 2 and 8 µg/mL in isolates. For some strains, the high colistin MIC observed was due to the presence of additional chromosomal mechanisms of resistance (Table 1). Interestingly, we observed that *K. pneumoniae* strains with high MIC appeared to lose *mcr-1* plasmids more easily. For these strains, the loss of the *mcr-1* gene did not lead to a change in their MIC, and they remained resistant to colistin. Hence, the *mcr-1* gene was not essential to their survival. In our study, the clonal dissemination of *E. coli* carrying the *mcr-1* gene was diverse, with 17 different STs, although some STs appeared several times. These STs were not related to a common sample origin or to a specific country. The presence of the same ST 4015 in two strains that were isolated in a same village from a human and a pig was described as a possible case of animal/human transmission [21]. The presence of ST 648 in two different travellers and ST 3997 in two villagers would appear to be a case of inter-human transmission. ST 48 and specially ST 10 were widespread STs, and have often been described for *mcr-1* in the literature [7]. Strains could carry different types of plasmids encoding the *mcr-1* gene [12,64]. Hence, the spread of the *mcr-1* gene could be unrelated to a specific clonal population.

Thus far, eight plasmid types carrying the *mcr-1* gene have been described around the world [12]. Our study confirmed this by observing only three plasmid types that were randomly present in different countries, as well as in humans and animals. *Mcr-1* database analysis confirms the global propagation of such plasmids, especially the smallest IncI2- and IncX4-type plasmids. The genetic environment of the *mcr-1* gene was first described by the IS*Apl1*-*mcr-1*-ORFcassette [6]. In this study, this combination was commonly found. The transposon Tn*6330* (IS*Apl1*-*mcr-1*-ORF- IS*Apl1*), described as being responsible for *mcr-1* gene transfer [15,58], was found in few strains, including *E. coli* LH30. This could be the reason for early *mcr-1* gene loss in this strain. Interestingly, the presence of the IS*Apl1*sequence was not always around the *mcr-1* gene [13,45]. In pLH1-mcr1, IS*Apl1* was found downstream of the *mcr-1* gene and inside the *traE* gene. This insertion sequence disrupts the *traE* gene, leading to the inactivation of this gene and affecting the capacity of the bacteria to transfer the plasmid through conjugation. This insertion site into the *traE* gene has already been described in such IncK2 plasmids in Switzerland, and has been associated with a lower frequency of conjugation [72,73].

A certain percentage of plasmids did not display an IS*Apl1* sequence around the *mcr-1* gene [16,74]. This led to two hypotheses: the *mcr-1* gene lost IS*Apl1* and stabilized it into a plasmid, or the *mcr-1* gene was transferred by other insertion sequences. The presence of IS*Apl1* in other places in the plasmid supports the first hypothesis and confirmed its original role in *mcr-1* gene transfer. Furthermore, in the composite transposon Tn*2706*, which is composed of 2 IS*30*, the loss of one IS was described as being favourable for the stabilization of genes in the new location [75].

In a recent study, IS*Apl1* was described as being a highly active IS and being able to transpose at a very high frequency in different nonspecific insertion sites [45]. For pLH1-mcr1, transposition was performed into a hotspot in the vicinity of a truncated IS1 insertion sequence. Moreover, a recombinase and a resolvase were found near the *mcr-1* transposon of p1RC4-mcr1 and pLH57-mcr1. Resolvase or recombinase are nucleases involved in DNA recombination. Their presence was a sign of recombination hotspots that were favourable to a transposon insertion. For pLH30-mcr1, an HNH endonuclease was present near the *mcr-1* transposon. Homing endonucleases were involved with lateral transfer as an intron to a homologous allele [76]. This could also be interpreted as a sign of a variable region suitable for insertion sequences.

## 5. Conclusions

Here, we show that worldwide dissemination of *mcr-1* encoding gene was due to the spread of a transposon, which can be found in different plasmid types and/or bacterial chromosomes. Even if some STs were redundant, dissemination was not due to a specific clonal population or to a specific plasmid type. Initially, the transfer of the *mcr-1* gene was due to Tn*6330*. Then, in order to stabilize in a new location, the transposon lost one or both IS*Apl1* sequences. It is likely that the evolution of the genetic environment of the *mcr-1* gene could lead to a diversity of insertion sequences of the *mcr-1* gene. This could raise the risk of a possible translocation into the chromosome. An emergence of a preponderant clone and the rapid dissemination of the *mcr-1* gene in Gram-negative bacteria are possible. Hence, we consider that it is essential to continue to survey resistance to colistin in those strains.

## Figures and Tables

**Table 1:** Origins and genotypic characteristics of *mcr-1* strains analysed in this study.

Strains *mcr-1*	Country	Origin	Other Known Colistin Mechanisms	MIC Colistin	ST	Plasmid Stability	Conjugation	Transformation	Plasmid Typing	IS*Apl1*
*Escherichia coli* LH1	Laos	Human		6	4015	+	-	-	IncHI2	downstream
*E. coli* LH30	Laos	Human		6	4012	−/− (0.25)	+	ND	IncHI2	down+upstream
*E. coli* LH57	Laos	Human	PhoQ mut (E375K)	8	3997	+	+	ND	IncHI2	down *+upstream *
*E. coli* LH121	Laos	Human		16	4013	+	+	ND	IncP	/
*E. coli* LH140	Laos	Human	PhoQ mut (E375K)	12	3997	+	+	ND	IncHI2	downstream
*E. coli* LH257	Laos	Human		12	4014	+	+	ND	IncI2	down+upstream
*E. coli* P10	Laos	Pig		6	4015	+	-	+	IncP	/
*E. coli* P6	Laos	Pig		6	4704	+	+	ND	IncI2	/
*E. coli* P17	Laos	Pig		4	93	+	-	+	IncI2	downstream
*E. coli* TH214	Thailand	Human		6	10	+	+	ND	IncI2	downstream
*E. coli* TH99	Thailand	Human		4	48	+	+	ND	IncI2	/
*E. coli* SE65	Algeria	Human		4	405	+	+	ND	IncP	downstream
*E. coli* 235	Algeria	Chicken		4	5758	+	+	ND	IncHI2	downstream
*E. coli* SA9	Algeria	Chicken		3	48	+	+	ND	IncHI2	downstream
*E. coli* SE3	Algeria	Chicken		3	48	+	+	ND	IncHI2	downstream
*E. coli* 1R	Traveler	Human		4	453	+	-	+	IncP	downstream
*E. coli* 6R	Traveler	Human		4	648	+	+	-	IncHI2	downstream
*E. coli* 44A	Traveler	Human		4	93	+	+	ND	IncHI2	/
*E. coli* 85R	Traveler	Human		4	656	+	+	ND	IncI2	down+upstream
*E. coli* 95R	Traveler	Human		4	10	+	+	ND	IncI2	downstream
*E. coli* 96R	Traveler	Human		4	10	+	+	ND	IncI2	downstream
*E. coli* 117R	Traveler	Human		4	648	+	+	ND	IncHI2	downstream
*E. coli* 1RC4	Traveler	Human		4	155	+	+	ND	IncHI2	down+upstream
*E. coli* 134R	Traveler	Human		3	602	+	+	ND	IncI2	downstream
*E. coli* 143R	Traveler	Human		3	1300	+	+	ND	IncHI2	downstream
*Klebsiella pneumoniae* 119R	Traveler	Human		3	788	+	+	ND	IncI2	downstream
*K. pneumoniae* LH131	Laos	Human	MgrB (stop)	32	1319	−/+ (32)	-	-	ND	/
*K. pneumoniae* LH17	Laos	Human	PmrB mut (T157P)	12	37	−/+ (12)	-	-	ND	/
*K. pneumoniae* LH61	Laos	Human	MgrB (substitution)	16	491	−/+ (16)	+	ND	IncI2	/
*K. pneumoniae* LH92	Laos	Human		12	39	−/− (12)	-	-	ND	/
*K. pneumoniae* FHM128	France	Human		4	1310	+	-	-	ND	downstream
*K. pneumoniae* FHA60	France	Human		8	1307	+	-	-	ND	downstream

* refers to partial sequences, MIC: Minimum Inhibitory Concentration, ST: Sequence Type, ND: Not Determined.

**Table 2:** IS*Apl1* presence, plasmid types and origins of *mcr-1* plasmids that were included in our database.

Genbank Accession Number	Type of Plasmid	GC%	Size (bp)	Strain *mcr-1*	Country	Origin	IS*Apl1* Presence in Plasmid	Reference
CP015913.1	IncI2	43.11	65,888	*Escherichia coli*	USA	Animal	Not present	[39]
CP015977.1	IncX4	41.85	33,304	*E. coli*	Brazil	Human	Not present	[40]
CP016183.1	IncHI1B	46.93	230,278	*E. coli*	Malaysia	Animal	Downstream *mcr-1*	[41]
CP016184.1	IncHI1B	47.04	235,403	*E. coli*	Malaysia	Animal	Downstream *mcr-1* + 1 other copy	[41]
CP016185.1	IncI2	42.48	61,735	*E. coli*	Malaysia	Animal	Not present	[41]
CP016186.1	IncI2	42.25	60,218	*E. coli*	Malaysia	Environment	Not present	[41]
CP016187.1	IncI2	42.35	60,950	*E. coli*	Malaysia	Animal	Not present	[41]
CP016405.1	IncI2	42.65	63,329	*E. coli*	USA	Animal	Not present	[42]
CP016550.1	IncX4	42.5	49,695	*E. coli*	Netherlands	Human	Not present	[39]
CP017246.1	IncX4	42.48	34,992	*E. coli*	Brazil	Animal	Not present	[43]
CP017632.1	IncN	47.4	369,298	*E. coli*	China	Human	Tn*6330* + 2 other copies	[44]
CP018106.1	IncI2	42.82	64,467	*E. coli*	Germany	Human	Downstream *mcr-1*	[45]
CP018112.1	IncI2	42.82	64,467	*E. coli*	USA	Human	Downstream *mcr-1*	[45]
CP018118.1	IncI2	42.82	64,467	*E. coli*	USA	Human	Downstream *mcr-1*	[45]
CP018124.1	IncI2	42.8	65,539	*E. coli*	USA	Human	Downstream *mcr-1* + 1 other copy	[45]
CP018773.1	IncX4	41.84	33,305	*E. coli*	USA	Human	Not present	[46]
KP347127.1	IncI2	43	64,015	*E. coli*	China	Animal	Downstream *mcr-1*	[6]
KU341381.1	IncHI2	46.53	251,493	*E. coli*	China	Animal	Downstream *mcr-1*	[6]
KU353730.1	IncFII	50.67	79,798	*E. coli*	Belgium	Animal	Not present	[47]
KU647721.2	IncX4	45.95	48,350	*E. coli*	Unknown	Animal	Not present	[48]
KU743383.1	IncX4	41.85	33,311	*E. coli*	Estonia	Animal	Not present	[49]
KU743384.1	IncHI2	46.21	240,367	*E. coli*	Saudi Arabia	Human	Tn*6330* + 1 other copy	[50]
KU761326.1	IncI2	42.65	64,964	*E. coli*	China	Human	Not present	[51]
KU761327.1	IncX4	41.84	33,287	*Klebsiella pneumoniae*	China	Human	Not present	[51]
KU870627.1	IncI2	42.46	62,219	*E. coli*	South Africa	Animal	Downstream *mcr-1*	[52]
KU922754.1	IncI2	42.3	57,059	*Kluyvera ascorbata*	China	Environment	Not present	[53]
KU934209.1	IncI2	42.26	65,419	*Salmonella enterica*	China	Animal	Downstream *mcr-1*	[54]
KU994859.1	IncFII	49.34	91,041	*E. coli*	Belgium	Animal	Downstream *mcr-1* + 1 other partial copy	[55]
KX013538.1	IncI2	42.43	61,228	*E. coli*	United Arab Emirates	Human	Downstream *mcr-1*	[50]
KX013539.1	IncI2	42.69	62,661	*E. coli*	Bahrain	Human	Not present	[50]
KX013540.1	IncI2	42.49	64,942	*E. coli*	Bahrain	Human	Not present	[50]
KX032519.1	IncI2	42.63	61,177	*E. coli*	South Africa	Human	Downstream *mcr-1*	[56]
KX032520.1	IncX4	41.9	31,808	*E. coli*	South Africa	Human	Not present	[56]
KX034083.1	IncI2	42.51	67,134	*E. coli*	China	Animal	Downstream *mcr-1*	[57]
KX084392.1	IncX4	41.85	33,298	*E. coli*	China	Animal	Not present	[58]
KX084393.1	IncI2	42.64	63,656	*E. coli*	China	Animal	Not present	[58]
KX084394.1	IncHI2	46.13	243,572	*E. coli*	China	Animal	Tn*6330*	[58]
KX129782.1	IncHI2	46.7	247,885	*E. coli*	Italy	Food	Tn*6330*	[59]
KX129783.1	IncX4	42.27	34,640	*E. coli*	Switzerland	Environment	Not present	[59]
KX129784.1	IncHI1B	46.48	209,401	*E. coli*	Thailand	Food	Not present	[59]
KX236309.1	IncX4	41.85	33,303	*K. pneumoniae*	Italy	Human	Not present	[60]
KX254341.1	IncHI2	46.64	267,486	*E. coli*	China	Animal	Not present	[58]
KX254342.1	IncI2	42.64	63,656	*E. coli*	China	Animal	Downstream *mcr-1*	[58]
KX254343.1	IncX4	41.84	33,307	*E. coli*	China	Animal	Not present	[58]
KX257480	IncFII	48.01	54,502	*S. enterica*	Unknown	Animal	Incomplete downstream *mcr-1*	[61]
KX257481	IncFII	47.99	54,670	*S. enterica*	Unknown	Animal	Incomplete downstream *mcr-1*	[61]
KX257482	IncFII	48.02	54,494	*S. enterica*	Unknown	Animal	Not present	[61]
KX276657.1	IncN	47.99	225,069	*E. coli*	USA	Human	Downstream *mcr-1* + 1 other copy	[62]
KX377410.1	IncFIB	46.94	57,278	*K. pneumoniae*	China	Environment	Downstream *mcr-1* + 1 other copy	[63]
KX447768.1	IncX4	41.84	33,395	*E. coli*	USA	Human	Not present	[64]
KX505142.1	IncI2	42.64	65,203	*Cronobacter sakazakii*	China	Animal	Downstream *mcr-1*	[31]
KX518745.1	IncY	47.46	97,559	*E. coli*	China	Animal	Tn*6630*	[65]
KX528699.1	IncFIB	46.99	15,998	*E. coli*	Vietnam	Animal	Downstream *mcr-1* + 1 other copy	[66]
KX570748.1	IncX4	41.96	32,751	*E. coli*	China	Animal	Not present	[67]
KX772391.1	IncFIB	49.97	179,444	*E. coli*	China	Human	Tn*6330*	[68]
KX772777.1	IncX4	41.84	33,309	*E. coli*	China	Human	Not present	[69]
KX772778.1	IncI2	42.47	65,375	*E. coli*	China	Human	Not present	[69]
KX880944.1	IncY	47.75	97,386	*E. coli*	China	Animal	Tn*6630*	[70]
LT174530	IncI2	42.5	61,826	*Shigella sonnei*	Vietnam	Human	Downstream *mcr-1*	[71]

**Table 3:** In silico analysis of the four sequences of *mcr-1* plasm.

*mcr-1* Plasmid	Type of Plasmid	pMLST	GC%	Size (bp)	Resistance Genes	IS*Apl1* Presence in Plasmid	Genbank Accession Number
pLH30-mcr1	IncHI2	ST 3	45.91	223,898	*mcr-1*, *bla*_TEM-217_, *cmlA1*, *floR*, *aph3-Ia*, *aadA2*, *sulIII*, *dfrA17*, *mefB*	2 sequences: 1 downstream and 1 upstream *mcr-1* gene	NKYL00000000
pLH57-mcr1	IncHI2	Unknown ST	48.00	218,800	*mcr-1*, *bla*_TEM-217_, *tetA*, *tetR*, *strA*, *strB*, *aph3’’-Ib*, *aph6-Id*, *sulII*, *dfrA14*	2 truncated sequences: 1 downstream and 1 upstream *mcr-1* gene	NKYM00000000
pLH1-mcr1	IncHI2	ST 3	46.38	248,201	*mcr-1*, *bla*_TEM-217_, *tetA*, *tetR*, *aadA2*, *oqxA*, *oqxBgb*, *sulI*, *sulIII*, *dfrA12*	2 sequences: 1 downstream *mcr-1* gene and 1 inside *traE* gene	NKYJ00000000
p1RC4-mcr1	IncHI2	ST 4	46.22	239,098	*mcr-1*, *bla*_TEM-217_, *tetA*, *tetR*, *floR*, *aph3-Ia*, *aph3-Ib*, *aadA1*, *strA*, *sulIII*, *dfrA14*	2 sequences: 1 downstream and 1 upstream *mcr-1* gene	NKYK00000000
